# Appointment reminders to increase uptake of HIV retesting by at‐risk individuals: a randomized controlled study in Thailand

**DOI:** 10.1002/jia2.25478

**Published:** 2020-04-15

**Authors:** Nicolas Salvadori, Pierrick Adam, Jean‐Yves Mary, Luc Decker, Lucie Sabin, Sylvie Chevret, Surachet Arunothong, Woottichai Khamduang, Prapan Luangsook, Visitsak Suksa‐ardphasu, Jullapong Achalapong, Christine Rouzioux, Wasna Sirirungsi, Nicole Ngo‐Giang‐Huong, Gonzague Jourdain

**Affiliations:** ^1^ Institut de recherche pour le développement (IRD)‐PHPT Marseille France; ^2^ Faculty of Associated Medical Sciences Chiang Mai University Chiang Mai Thailand; ^3^ Department of Epidemiology, Biostatistics and Occupational Health McGill University Montreal Canada; ^4^ INSERM U1153 Team ECSTRA Université Paris Diderot ‐ Paris 7 Hôpital Saint‐Louis Paris France; ^5^ Department of Disease Control Ministry of Public Health Chiang Mai Thailand; ^6^ Chiangrai Prachanukroh Hospital Chiang Rai Thailand; ^7^ Laboratoire de Virologie ‐ EA 3620 Université Paris Descartes Hôpital Necker Paris France; ^8^ Department of Immunology and Infectious Diseases Harvard T.H. Chan School of Public Health Boston MA USA

**Keywords:** reminder, appointment, text messaging, cell phone, testing, retesting

## Abstract

**Introduction:**

Frequent HIV testing of at‐risk individuals is crucial to detect and treat infections early and prevent transmissions. We assessed the effect of reminders on HIV retesting uptake.

**Methods:**

The study was conducted within a programme involving four facilities providing free‐of‐charge HIV, syphilis and hepatitis B and C testing and counselling in northern Thailand. Individuals found HIV negative and identified at risk by counsellors were invited to participate in a three‐arm, open‐label, randomized, controlled trial comparing: (a) “No Appointment & No Reminder” (control arm); (b) “No Appointment but Reminder”: short message service (SMS) sent 24 weeks after the enrolment visit to remind booking an appointment, and sent again one week later if no appointment was booked; and (c) “Appointment & Reminder”: appointment scheduled during the enrolment visit and SMS sent one week before appointment to ask for confirmation; if no response: single call made within one business day. The primary endpoint was a HIV retest within seven months after the enrolment visit. The cost of each reminder strategy was calculated as the sum of the following costs in United States dollars (USD): time spent by participants, counsellors and hotline staff; phone calls made; and SMS sent. The target sample size was 217 participants per arm (651 overall).

**Results:**

Between April and November 2017, 651 participants were randomized. The proportion presenting for HIV retesting within seven months was 11.2% (24/215) in the control arm, versus 19.3% (42/218) in “No Appointment but Reminder” (*p* = 0.023) and 36.7% (80/218) in “Appointment & Reminder” (*p* < 0.001). Differences in proportions compared to the control arm were respectively +8.1% (95% CI: +1.4% to +14.8%) and +25.5% (+17.9% to +33.2%). The incremental cost‐effectiveness ratios of “No Appointment but Reminder” and “Appointment & Reminder” compared to the control arm were respectively USD 0.05 and USD 0.14 per participant for each 5% increase in HIV retesting uptake within seven months.

**Conclusions:**

Scheduling an appointment and sending a reminder one week before was a simple, easy‐to‐implement and affordable intervention that significantly increased HIV retesting uptake in these at‐risk individuals. The personal phone call to clients probably contributed, and also improved service efficiency.

## 1 Introduction

Ensuring that individuals at risk of HIV infection are frequently tested is essential to treat new infections as early as possible after virus acquisition and, thus, prevent new transmissions. However, in 2018, only 79% of people living with HIV worldwide knew their status [1] and 1.7 million new HIV infections occurred [2].

After a generalized HIV epidemic which started in the late 1980s, Thailand is now facing an epidemic mainly concentrated in key populations, in particular in men who have sex with men [3]. In 2018, Thailand reported that it achieved the first UNAIDS 90% target [1, 4]. However, it also reported that 53% of newly diagnosed individuals had a CD4 cell count <200 cells/mm^3^ at diagnosis and that 18,000 people died of AIDS‐related causes [3], suggesting that HIV was often diagnosed too late. In this context, the Thai government is supporting all initiatives to increase access to HIV testing and linkage to care.

The 2019 World Health Organization (WHO) guidelines on HIV testing services suggested a wide range of approaches to increase demand for HIV testing services [5] but no specific strategies to retain at‐risk individuals into HIV prevention/sexual health programmes – with or without pre‐exposure prophylaxis – notably those involving reminders. The wide availability of mobile phones (8.2 billion subscriptions worldwide in 2018, including 6.5 billion in developing countries [6]) may provide opportunities to improve health outcomes due to their low cost [7], convenience and variety of communication means (calls, voice messages, short message service (SMS), mobile applications). For example, SMS‐based interventions have been found effective in improving adherence to antiretroviral therapy in HIV‐infected people [8] and WHO recommend their use in this context [7]. SMS reminders also have the potential to increase the uptake of frequent HIV testing in uninfected at‐risk individuals [9]. However, previous studies had limitations: they either were observational [10, 11, 12] or assessed the effect of sending SMS reminders to retest participants presenting with a suspected acute HIV infection one month after a first negative test, a very specific situation [13].

We conducted a randomized controlled trial in Thailand to evaluate whether reminders could increase the uptake of HIV retesting by at‐risk individuals.

## 2 Methods

### 2.1 Study setting

“Napneung” was designed as a research project aimed at evaluating new methods to increase the frequency of HIV testing by at‐risk individuals [14]. Trained nurses and medical technologists provided free‐of‐charge anonymous and confidential testing and counselling in four settings in Chiang Mai and Chiang Rai, two medium‐sized cities in northern Thailand. All individuals aged 15 years or above were welcome for testing and counselling, regardless of gender, sexual orientation, risk behaviour or history of HIV testing. Outreach of at‐risk individuals relied on distribution of vouchers in public places, posters, social media and digital advertising campaigns. After an appointment made through a 24/7 telephone line or online through the project website, clients were provided with pre‐ and post‐test counselling and rapid testing for HIV, syphilis, hepatitis B surface antigen and hepatitis C antibodies in less‐than‐one‐hour sessions. While waiting for the test results, clients were invited to complete self‐administered questionnaires about their sociodemographic and behavioural characteristics on a tablet computer. All clients were provided with a unique study identification card and a secret code so that those presenting for retesting could be linked to previously recorded data. No incentives were provided for presenting for retesting.

### 2.2 Study design and population

Within the Napneung project, we implemented a three‐arm randomized controlled study to evaluate and compare the effect of scheduling appointments and sending reminders on the uptake of HIV retesting among at‐risk individuals (ClinicalTrials.gov: NCT02752152). This study was nested within another randomized study designed to compare the efficacy of three counselling methods in terms of propensity to present for retesting [14].

Clients were eligible to participate in the study if they were aged 18 years or above, tested HIV negative and reported risks of HIV infection. Guidelines used to consider that individuals at risk of HIV acquisition were those from key populations [15]. However, the definition of an individual's risk and its assessment are difficult. In this study, we decided to rely on counsellors, who were trained to base their assessment on clients' self‐reported behaviours during the last six months and to be cautious about intention‐behaviour gaps [16]. This operational definition is closer to the 2019 WHO guidelines, which recommend HIV retesting at least annually in people who have ongoing HIV‐related risks [5].

Consenting participants were randomly assigned 1:1:1 to one of the following three arms:

- “No Appointment & No Reminder” (control arm): at the enrolment visit, the counsellor encouraged the client to present for HIV retesting within three to six months or even within less than three months if the perceived risk was high; then, no further contact was made;
- “No Appointment but Reminder”: at the enrolment visit, the counsellor encouraged the client to present for HIV retesting within three to six months or even within less than three months if the perceived risk was high; 24 weeks later, the following SMS was sent: *“Time to visit Napneung again! Please make an appointment at [hotline phone number]”*; if no appointment was booked within one week, the same SMS was sent again once; or
- “Appointment & Reminder”: at the enrolment visit, the counsellor and the client agreed on a date and time for the next HIV test, within three to six months or even within less than three months if the perceived risk was high; one week before the scheduled appointment, the following SMS was sent: *“Napneung: appointment on [date and time] at [testing facility]. Please reply 'Yes' to confirm”*; one business day later, if the client did not reply to the SMS, one phone call was made by our hotline staff to clarify the appointment status.

Randomization was performed with a block size of six and stratified per counsellor and by counselling method received as part of the three‐arm counselling study.

### 2.3 Reminders

All participants randomized to arms “No Appointment but Reminder” or “Appointment & Reminder” were invited to provide a mobile phone number. Those who did not provide a phone number or did not agree for the use of their phone number to receive reminders were eligible to participate in the study but were not sent any reminders. Mobile phone numbers and all participant data were collected anonymously on tablet computers, encrypted, transmitted and stored in real‐time within a central database hosted on a secured server with restricted access and extended traceability features. Verification of mobile phone numbers was performed and documented immediately after entry by sending a test SMS to participants. Reminders for retesting were only sent to participants who agreed for the use of their phone number to receive reminders. SMS were sent at 5.45pm every day by an automated system according to criteria and characteristics recorded in the study database, including participant’s preferred language (Thai, Burmese, Shan or English). Reminders were not sent to participants who presented for retesting earlier than scheduled. Phone calls were made by the project hotline staff between 4pm and 8pm. After the first retest visit (if any), participants were offered the same reminder procedures for further retest visits until the end of the study follow‐up period (31 January 2019), unless opting out.

### 2.4 Endpoints

The primary endpoint was a HIV retest at any of the study facilities within seven months after the enrolment visit, and the secondary endpoint was a HIV retest at any of the study facilities within 12 months.

### 2.5 Sample size calculation

We assumed that 25% of participants in the control arm and 40% in each experimental arm would present for HIV retesting within seven months after the enrolment visit. One interim analysis for efficacy was planned when half of participants have reached the primary endpoint assessment timepoint, but this analysis was not performed because the target accrual for the final analysis had already been reached by that time. The overall two‐sided type I error for pairwise comparisons between the control arm and each experimental arm was set to 0.001 for the interim analysis and 0.049 for the final analysis, with adjustment for multiple comparisons using Sidak correction, and the power was set to 90%. Based on these parameters and Fisher's exact tests, the final analysis was planned when 217 participants per arm, that is, 651 overall, reached the primary endpoint assessment timepoint.

### 2.6 Statistical analyses

For each arm, we calculated the proportions of participants presenting for retesting within seven months and within twelve months after enrolment and their 95% confidence intervals (CI) using the Clopper‐Pearson method. Comparisons of these proportions between each experimental arm and the control arm were performed using two‐sided Fisher’s exact tests. The effect of each experimental arm relative to the control arm was expressed as a difference in proportions, with 95% CI based on the normal approximation to the binomial distribution. We assessed whether the effect of the reminder methods on HIV retesting uptake within seven months was different across counselling methods by testing the interaction between the effects of reminder and of counselling methods on HIV retesting uptake in a logistic regression model (more details are provided in the footnotes of Table S1). A comparison between the two experimental arms was performed in a post hoc analysis.

### 2.7 Intervention costs and cost‐effectiveness

We calculated the total cost of each reminder strategy as the sum of the following costs (where applicable): time spent by participants, counsellors and hotline staff; phone calls made; and SMS sent. Cost of time spent by participants was estimated based on actual time spent with study staff and on the monthly income that they reported in the self‐administered questionnaire or, if null or missing, on an imputed monthly income corresponding to the minimum wage in Chiang Mai. Cost of time spent by counsellors and hotline staff was estimated based on actual time spent with participants and on their monthly income. We estimated the main fixed costs, but excluded them from the cost calculations because the information technology (IT) infrastructure to send SMS reminders could have been used for many more clients than those who participated in this study [17]. All costs were adjusted for the consumer price index as of 2015 and converted from Thai Baht (THB) to United States dollars (USD) at the yearly average exchange rate for 2015 (THB 1 = USD 0.02919). The incremental cost‐effectiveness ratio of each experimental arm compared to the control arm was calculated as the difference in mean costs per participant between these two strategies divided by the difference in proportions of participants presenting for retesting within seven months between these two strategies.

### 2.8 Ethical considerations

All procedures performed in this study were in accordance with the Declaration of Helsinki. Informed consent was obtained from all individual participants included in the study. The study protocol was reviewed and approved by the ethics committees of the Faculty of Associated Medical Sciences, Chiang Mai University, and of Chiangrai Prachanukroh Hospital. An Advisory Committee reviewed the study progress annually. In addition, the study was reviewed and discussed quarterly by a Community Advisory Board.

## 3 Results

### 3.1 Study population

**Figure 1:**
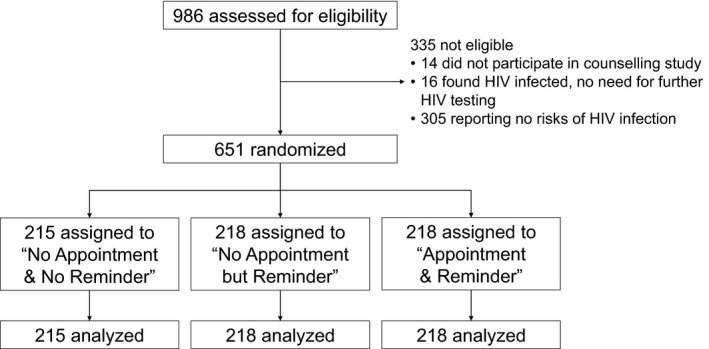
CONSORT diagram of the disposition of participants.

Between 25 April 2017 and 1 December 2017, a total of 651 participants were randomized: 215 to “No Appointment & No Reminder,” 218 to “No Appointment but Reminder” and 218 to “Appointment & Reminder” (Figure 1). The distribution of participants’ characteristics and counselling arm was similar between arms (Table 1). Of the 651 participants, 359 (55%) were men, median age was 24 (interquartile range (IQR), 22 to 31) years, 401 (63%) had never been tested for HIV, 236 (37%) had at least two sexual partners in the last three months and 261 (41%) used condoms inconsistently in the last three months. No protocol deviations occurred during the study.

**Table 1:** Participants' characteristics by arm

Participants' characteristics	No appointment & no reminder (N = 215)	No appointment but reminder (N = 218)	Appointment & reminder (N = 218)	Overall
Gender				
Male	123 (57%)	120 (55%)	116 (53%)	359 (55%)
Female	91 (42%)	98 (45%)	99 (45%)	288 (44%)
Male‐to‐female transgender	1 (<1%)	0 (0%)	3 (1%)	4 (1%)
Age <25 years	124 (58%)	131 (60%)	122 (56%)	377 (58%)
Country				
Thailand	211 (98%)	213 (98%)	214 (98%)	638 (98%)
Myanmar	4 (2%)	2 (1%)	3 (1%)	9 (1%)
Other	0 (0%)	3 (1%)	1 (<1%)	4 (1%)
Pursued higher education	131/212 (62%)	129/214 (60%)	130/217 (60%)	390/643 (61%)
Man who has ever had sex with men	57 (27%)	54 (25%)	42 (19%)	153 (24%)
Previously tested for HIV	84/209 (40%)	79/213 (37%)	73/215 (34%)	236/637 (37%)
Counselling method received				
Standard counselling	89 (41%)	86 (39%)	90 (41%)	265 (41%)
Computer‐assisted counselling	88 (41%)	93 (43%)	88 (40%)	269 (41%)
On‐demand counselling	38 (18%)	39 (18%)	40 (18%)	117 (18%)
Agreed timeline for retesting				
Within less than three months after enrolment visit	22 (10%)	25 (11%)	25 (11%)	72 (11%)
Within three to six months after enrolment visit	193 (90%)	193 (89%)	193 (89%)	579 (89%)
Risks of HIV infection				
Inconsistent condom use in last three months	80/209 (38%)	88/213 (41%)	93/213 (44%)	261/635 (41%)
≥2 sexual partners in last three months	83/208 (40%)	78/211 (37%)	75/212 (35%)	236/631 (37%)
Positive syphilis test	12 (6%)	5 (2%)	6 (3%)	23 (4%)
STI symptoms	8/207 (4%)	7/205 (3%)	9/207 (4%)	24/619 (4%)
Ever had an STI	30/208 (14%)	17/214 (8%)	22/212 (10%)	69/634 (11%)
Ever received benefits in exchange of sex	9/208 (4%)	6/210 (3%)	15/207 (7%)	30/625 (5%)
Ever provided benefits in exchange of sex	14/209 (7%)	18/210 (9%)	15/211 (7%)	47/630 (7%)
Ever had sex outdoors	30/211 (14%)	29/212 (14%)	23/211 (11%)	82/634 (13%)
Ever injected drugs	6/214 (3%)	5/215 (2%)	8/216 (4%)	19/645 (3%)

### 3.2 HIV retesting

**Figure 2:**
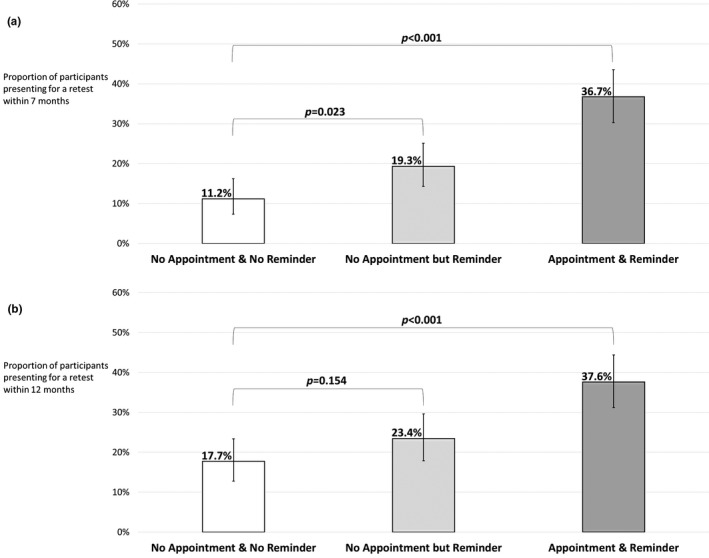
Participants retested for HIV within **(a)** seven months **(b)** twelve months by arm. “No Appointment & No Reminder”: clients encouraged to present for HIV retesting within three to six months or even within less than three months if the perceived risk was high, then no further contact made; “No Appointment but Reminder”: short message service (SMS) sent 24 weeks after the enrolment visit to remind booking an appointment, and sent again one week later if no appointment was booked; “Appointment & Reminder”: appointment scheduled during the enrolment visit and SMS sent one week before appointment to ask for confirmation; if no response: single call made within one business day. Bars represent 95% confidence intervals calculated using the Clopper–Pearson method. The overall two‐sided type I error for the final analysis was set to 0.049 for pairwise comparisons between the control arm and each experimental arm, that is, 0.0248 per comparison using Sidak correction. *p*‐values were derived from two‐sided Fisher’s exact tests. In a post hoc analysis, the *p*‐value for the comparison between the two experimental arms was <0.001 **(a)** 0.002 **(b)**.

In the primary analysis at seven months, the proportion of participants who had presented for HIV retesting was 11.2% (24/215; 95% CI: 7.3% to 16.2%) in “No Appointment & No Reminder,” as compared to 19.3% (42/218; 95% CI: 14.3% to 25.1%) in “No Appointment but Reminder” (*p* = 0.023) and to 36.7% (80/218; 95% CI: 30.3% to 43.5%) in “Appointment & Reminder” (*p* < 0.001) (Figure 2a). Differences in proportions compared to the control arm were respectively +8.1% (95% CI: +1.4% to +14.8%) and +25.5% (95% CI: +17.9% to +33.2%). In a post hoc analysis comparing the two experimental arms, the *p*‐value was <0.001 and the difference in proportions was +17.4% (95% CI: +9.2% to +25.7%). No interaction was observed between reminder and counselling methods (Table S1).

In the secondary analysis at 12 months, the proportion of participants who had presented for HIV retesting was 17.7% (38/215; 95% CI: 12.8% to 23.4%) in “No Appointment & No Reminder,” as compared to 23.4% (51/218; 95% CI: 17.9% to 29.6%) in “No Appointment but Reminder” (*p* = 0.15) and to 37.6% (82/218; 95% CI: 31.2% to 44.4%) in “Appointment & Reminder” (*p* < 0.001) (Figure 2b). In a post hoc analysis comparing the two experimental arms, the *p*‐value was 0.002. Of note, while no participant tested HIV positive within the first seven months, one in “No Appointment & No Reminder” tested positive between seven and twelve months.

By the end of the study follow‐up period, that is, within a median of 17.8 (IQR, 16.2 to 19.8) months after the enrolment visit, the proportions of participants who had presented for HIV retesting were 21.9% (47/215) in “No Appointment & No Reminder,” 26.2% (57/218) in “No Appointment but Reminder” and 37.6% (82/218) in “Appointment & Reminder.”

### 3.3 Delivery and outcome of retest reminders in experimental arms

A detailed description of delivery and outcome of retest reminders within the seven months after the enrolment visit is provided in Figures S1 and S2.

Of the 218 participants in arm “No Appointment but Reminder,” 28 (13%) called for a new appointment before receiving the SMS reminders (including four who did not receive them due to technical issues), 6 (3%) after receiving the first SMS and 8 (4%) after receiving the second SMS, and 176 (81%) did not call.

Of the 218 participants in arm “Appointment & Reminder,” 4 (2%) confirmed the retest appointment despite not receiving reminders due to technical issues, 17 (8%) confirmed it by SMS reply (13 on the day of SMS receipt (median of 47 (IQR, 13 to 96) minutes after), 3 on the following day and 1 two days later), 59 (27%) confirmed it after receiving the subsequent phone call, and 138 (63%) did not confirm it. Of the 80 participants who presented for HIV retesting within seven months, 37 presented on the same day as originally scheduled at the enrolment visit, 16 earlier than scheduled (median of 2 (IQR, 1 to 5) days before) and 27 later than scheduled (median of 6 (IQR, 2 to 9) days after).

Of the 401 participants who were sent SMS reminders, 125 (31%) did not receive them, for the following reasons (several possible): SMS blocked by the participant's mobile operator (n = 82), participant's mobile phone number no longer existing (n = 30) or gateway error (n = 14).

### 3.4 Intervention costs and cost‐effectiveness

We estimated that the total fixed cost was about USD 15,000, including developing the IT infrastructure, developing an in‐house software to send SMS messages and the protocol for SMS delivery, and others. The mean cost per participant of the reminder strategies was USD 0.15 for “No Appointment & No Reminder,” USD 0.22 for “No Appointment but Reminder” and USD 0.84 for “Appointment & Reminder,” mostly reflecting the costs associated with time spent by participants and study staff (Table 2). The incremental cost‐effectiveness ratios of “No Appointment but Reminder” and “Appointment & Reminder” compared to “No Appointment & No Reminder” were respectively USD 0.05 and USD 0.14 per participant for each 5% increase in uptake of HIV retesting within seven months, and that of “Appointment & Reminder” compared to “No Appointment but Reminder” was USD 0.18.

**Table 2:** Details of mean costs per participant for each reminder strategy from a societal perspective, in USD

Mean costs per participant	No appointment & no reminder (N = 215)	No appointment but reminder (N = 218)	Appointment & reminder (N = 218)
Costs borne by participants	USD 0.09	USD 0.10	USD 0.19
Time spent^a^	USD 0.03	USD 0.03	USD 0.17
Phone calls made	USD 0.06	USD 0.07	USD 0.01
SMS sent	None	None	USD 0.01
Counsellor‐related costs	None	None	USD 0.35
Time spent^b^	None	None	USD 0.35
Hotline staff related costs	USD 0.06	USD 0.12	USD 0.30
Time spent^b^	USD 0.06	USD 0.08	USD 0.17
Phone calls made	None	None	USD 0.08
SMS sent	None	USD 0.04	USD 0.05
Total mean cost per participant	USD 0.15	USD 0.22	USD 0.84

All costs were adjusted for the consumer price index as of 2015 and converted from THB to USD at the yearly average exchange rate for 2015 (THB 1 = USD 0.02919). SMS, short message service; USD, United States dollars.

^a^ Cost of time spent by participants was estimated based on actual time spent with study staff and on the monthly income that they reported in the self‐administered questionnaire or, if null or missing, on an imputed monthly income corresponding to the minimum wage in Chiang Mai (USD 280.22);

^b^ Cost of time spent by counsellors and hotline staff was estimated based on actual time spent with participants and on their monthly income.

## 4 Discussion

In this randomized trial, we found that individuals at risk of HIV infection were significantly more likely to present for HIV retesting within seven months after a first visit if they had an appointment scheduled during their first visit and were reminded of it (37%) or received no appointment but a reminder (19%) than if they received no appointment and no reminder (11%).

Uptake of HIV retesting in the arm where no appointment was scheduled in advance but an SMS reminder was sent (followed by a second one a week later if no appointment was made) was significantly higher than in the control arm, but remained low. Sending SMS reminders had a significant but limited positive effect, increasing from 11% to 19% the proportion of clients presenting for HIV retesting. Interestingly, the proportion of those who called for an appointment before receiving any SMS reminder was 13% (28/218), consistent with the 11% observed in the arm with no intervention. Sending one SMS reminder had little additional effect (3%; 6/218), and a second SMS reminder one week later as well (4%; 8/218). Using phone call reminders may have been more effective than sending SMS reminders to increase HIV retesting uptake.

The most active strategy, that is, when an appointment was scheduled in advance and a reminder sent, was the most successful. The higher success of this strategy compared to the other experimental strategy may be partly because reminders were sent earlier, the content of the SMS reminder was more personalised or calling participants was more effective than SMS [12] although, by design, the specific contribution of these components of the intervention could not be assessed. Interestingly, only 8% (17/218) confirmed the appointment by SMS reply, while 27% (59/218) confirmed it after receiving the subsequent phone call. This finding suggests that the provider's proactive and personalised attitude was a key component explaining the success of this intervention. One additional advantage of the most successful strategy is that after calling the participant, the hotline officer recorded immediately in the system whether the retest appointment was confirmed or cancelled. Therefore, the counsellors knew the status of the appointment several days prior to the scheduled appointment and managed their time more efficiently than in the other two reminder strategies.

Nearly one third of the clients who were sent SMS reminders did not receive them: many SMS were automatically blocked by mobile operators, a common issue with the use of automated SMS sending systems. Sending manually SMS reminders after notification that the automated SMS was blocked may add significant cost. Some mobile phone numbers were no longer existing, suggesting that some clients changed their phone number. Implementing phone call reminders in settings where changing mobile phone numbers is frequent may therefore be challenging. Sending reminders through instant messaging applications may be an alternative but a significant part of young people cannot afford mobile Internet access.

Results from the cost analysis indicate that the two experimental reminder strategies were affordable, each with a total mean cost per client of less than USD 1. Both strategies were highly cost‐effective in terms of HIV retesting uptake. Compared to the control arm, the additional mean cost per participant to increase by 5% the uptake of HIV retesting within seven months was less than USD 0.15, showing that the costs associated with these strategies are not a limitation for implementation. Indeed, we did not include fixed costs in the cost calculations, but one can assume that even a costly software developed at a national level for all HIV testing facilities would remain highly cost‐effective.

Strengths of our study were the rigorous methodology in a setting with experience in clinical research implementation, the appropriateness of the study population and the absence of bias that we could suspect. A limitation was that we were not able to know whether clients presented for HIV retesting in other facilities. However, there is no reason to expect a difference between arms in the proportion of such clients. This proportion was probably small given the high satisfaction rate reported at the first visit [14]. Another limitation was that the selection of study participants was based on a risk assessment performed by counsellors, which could vary across counsellors. However, randomization was stratified per counsellor.

## 5 Conclusions

Scheduling an appointment and sending a reminder one week before was a simple, easy‐to‐implement and affordable intervention that significantly increased the uptake of HIV retesting in this population of at‐risk individuals. The personal phone call to clients probably contributed, and also improved service efficiency. This intervention can be implemented at country level – including in resource‐limited settings – and has the potential to increase the retention of at‐risk individuals into HIV prevention services.

## Competing interest

The authors declare no competing interest.

## Authors' Contributions

NS, PA, JYM, LD, SC and GJ conceived and designed the study. LD created the IT tools. NS and LS analysed the data. NS, PA, JYM, LD, LS, SC, SA, WK, PL, VS, JA, CR, WS, NNGH and GJ interpreted the data. NS drafted the manuscript and all other authors revised the manuscript. All authors have read and approved the final manuscript.

## Supporting information


**Figure S1.** Delivery and outcome of retest reminders for the 218 participants in “No Appointment but Reminder.”

**Figure S2.** Delivery and outcome of retest reminders for the 218 participants in “Appointment & Reminder.”

**Table S1.** Participants retested for HIV within seven months by combination of counselling and reminder methods
