# Supplementary figures and images for: Appointment reminders to increase uptake of HIV retesting by at‐risk individuals: a randomized controlled study in Thailand
Source: J Int AIDS Soc. 2020 Apr 15;23(4):e25478. doi: 10.1002/jia2.25478 (PMC7159062; doi:10.1002/jia2.25478)

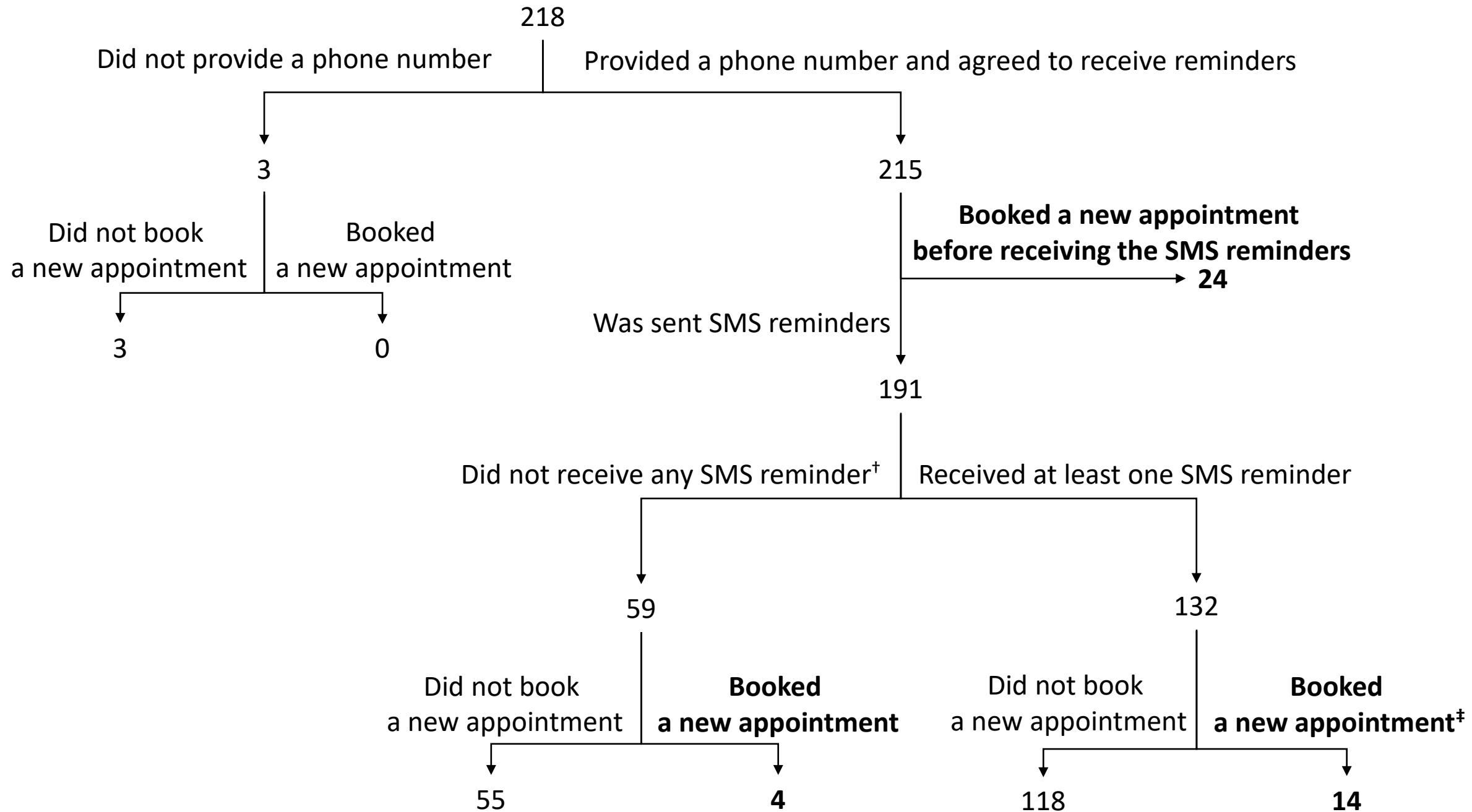

Supplement: Supplementary file 1 — Figure S1. Delivery and outcome of retest reminders for the 218 participants in “No Appointment but Reminder.” [file JIA2-23-e25478-s001.pdf]

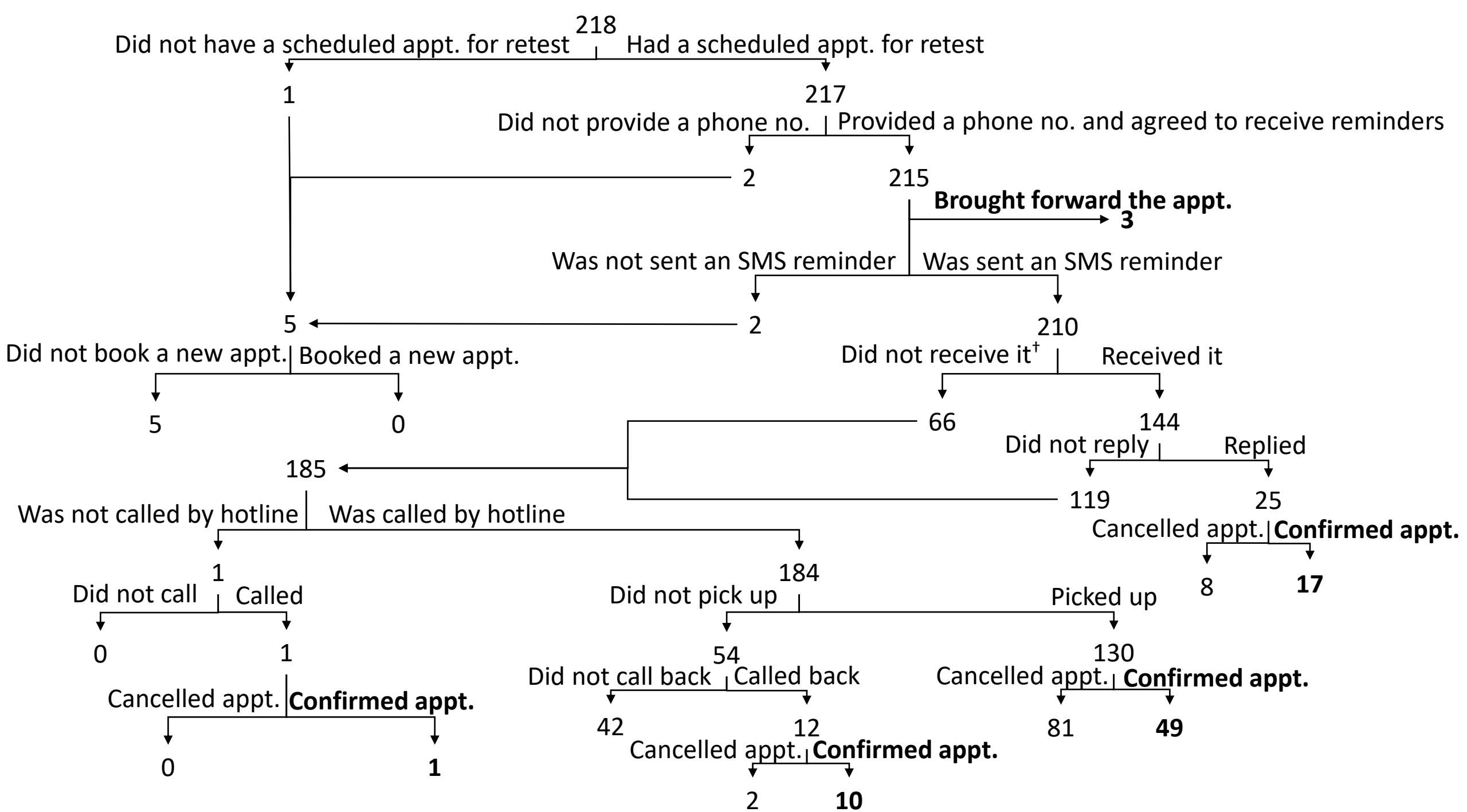

Supplement: Supplementary file 2 — Figure S2. Delivery and outcome of retest reminders for the 218 participants in “Appointment & Reminder.” [file JIA2-23-e25478-s002.pdf]
