# Supplementary material for: Appointment reminders to increase uptake of HIV retesting by at‐risk individuals: a randomized controlled study in Thailand
Source: J Int AIDS Soc. 2020 Apr 15;23(4):e25478. doi: 10.1002/jia2.25478 (PMC7159062; doi:10.1002/jia2.25478)
Supplement: Supplementary file 3 — Table S1. Participants retested for HIV within seven months by combination of counselling and reminder methods [file JIA2-23-e25478-s003.pdf]

	No Appointment & No Reminder	No Appointment but Reminder			Appointment & Reminder		
**Standard counseling**	11/89 (12%); Ref.	18/86 (21%); OR = 1.9	p=0.90	p=0.72	29/90 (32%); OR = 3.4	p=0.40	p=0.36
**Computer-assisted counseling**	10/88 (11%); Ref.	17/93 (18%); OR = 1.7			36/88 (41%); OR = 5.4		
**On-demand counseling**	3/38 (8%); Ref.	7/39 (18%); OR = 2.6			15/40 (38%); OR = 7.0		
